# *Tinospora crispa* (L.) Hook. f. & Thomson: A Review of Its Ethnobotanical, Phytochemical, and Pharmacological Aspects

**DOI:** 10.3389/fphar.2016.00059

**Published:** 2016-03-21

**Authors:** Waqas Ahmad, Ibrahim Jantan, Syed N. A. Bukhari

**Affiliations:** Drug and Herbal Research Centre, Faculty of Pharmacy, Universiti Kebangsaan MalaysiaKuala Lumpur, Malaysia

**Keywords:** *Tinospora crispa*, traditional uses, phytochemistry, pharmacological activities, toxicity studies, clinical trials

## Abstract

*Tinospora crispa* (L.) Hook. f. & Thomson (Menispermaceae), found in the rainforests or mixed deciduous forests in Asia and Africa, is used in traditional medicines to treat numerous health conditions. This review summarizes the up-to-date reports about the ethnobotany, phytochemistry, pharmacological activities, toxicology, and clinical trials of the plant. It also provides critical assessment about the present knowledge of the plant which could contribute toward improving its prospect as a source of lead molecules for drug discovery. The plant has been used traditionally in the treatment of jaundice, rheumatism, urinary disorders, fever, malaria, diabetes, internal inflammation, fracture, scabies, hypertension, reducing thirst, increasing appetite, cooling down the body temperature, and maintaining good health. Phytochemical analyses of *T. crispa* revealed the presence of alkaloids, flavonoids, and flavone glycosides, triterpenes, diterpenes and diterpene glycosides, *cis* clerodane-type furanoditerpenoids, lactones, sterols, lignans, and nucleosides. Studies showed that the crude extracts and isolated compounds of *T. crispa* possessed a broad range of pharmacological activities such as anti-inflammatory, antioxidant, immunomodulatory, cytotoxic, antimalarial, cardioprotective, and anti-diabetic activities. Most pharmacological studies were based on crude extracts of the plant and the bioactive compounds responsible for the bioactivities have not been well identified. Further investigations are required to transform the experience-based claims on the use of *T. crispa* in traditional medicine practices into evidence-based information. The plant extract used in pharmacological and biological studies should be qualitatively and quantitatively analyzed based on its biomarkers. There should be detail *in vitro* and *in vivo* studies on the mechanisms of action of the pure bioactive compounds and more elaborate toxicity study to ensure safety of the plant for human use. More clinical trials are encouraged to be carried out if there are sufficient preclinical and safety data.

## Introduction

Herbs are the sources of crude drugs that are used to treat pathologic conditions, often chronic in nature, or to achieve or retain a state of improved health. Several cultures have distinct uses of plants for the treatment of various diseases (Wyk and Wink, 2004). This traditional knowledge has been vocally passed on through a number of generations; therefore these traditional remedies are still in practice. This knowledge on traditional medical practice, collected over the centuries by trial and error using the patient as the experimental animal throughout, must contain some material worthy of additional research. This, consequently, calls to carry out scientific studies on such plants to confirm the claims of community folks on their medicinal effects.

*Tinospora crispa* (L.) Hook. f. & Thomson is a medicinal plant belongs to the genus *Tinospora* of Menispermaceae family. It is prevalent in primary rainforests or mixed deciduous forests of South East Asia and Africa including Thailand, Malaysia, and Indonesia (Pathak et al., 1995). It has been used in conventional medicine to treat numerous pathologies in Malaysia (Najib Nik a Rahman et al., 1999), Indonesia (Dweck and Cavin, 2006), Thailand (Kongsaktrakoon et al., 1984), and the Philippines (Quisumbing, 1951). There was a previous review of the secondary metabolites and biological activities of *T. crispa* (Koay and Amir, 2013), however, critical assessment of the present knowledge is needed to provide the perspectives and directions for future research and potential applications. The purpose of this review is to provide an updated and complete overview of the botany, phytochemistry, traditional uses, and pharmacological activities of *T. crispa*. Moreover, the present knowledge obtained mainly from experimental studies was critically assessed to provide evidences and justifications for local and traditional uses of *T. crispa* and to propose future research prospects and potential therapeutic uses for this plant.

## Vernacular names

*T. crispa*, is known as “Patawali,” “Akar Patawali,” “Seruntum,” or “Akar Seruntum” in Malaysia (Noor et al., 1989), “Brotawali,” “Antawali,” and “Andawali” in Indonesia (Roosita et al., 2008; Koay and Amir, 2013), “Makabuhay” (meaning “You may live”) in Philippines, (Quisumbing, 1951), “Boraphet” in Thailand, “Da ye ruanjinteng” in China (Li et al., 2006), “Banndol Pech” in Cambodia (Hout et al., 2006) “Guloncho-ban” or “Golonchi” in Bangladesh (Rahmatullah et al., 2011), and “Lyann span Zeb kayenn” in Martinique island (Longuefosse and Nossin, 1996).

## Plant description

**Figure 1:**
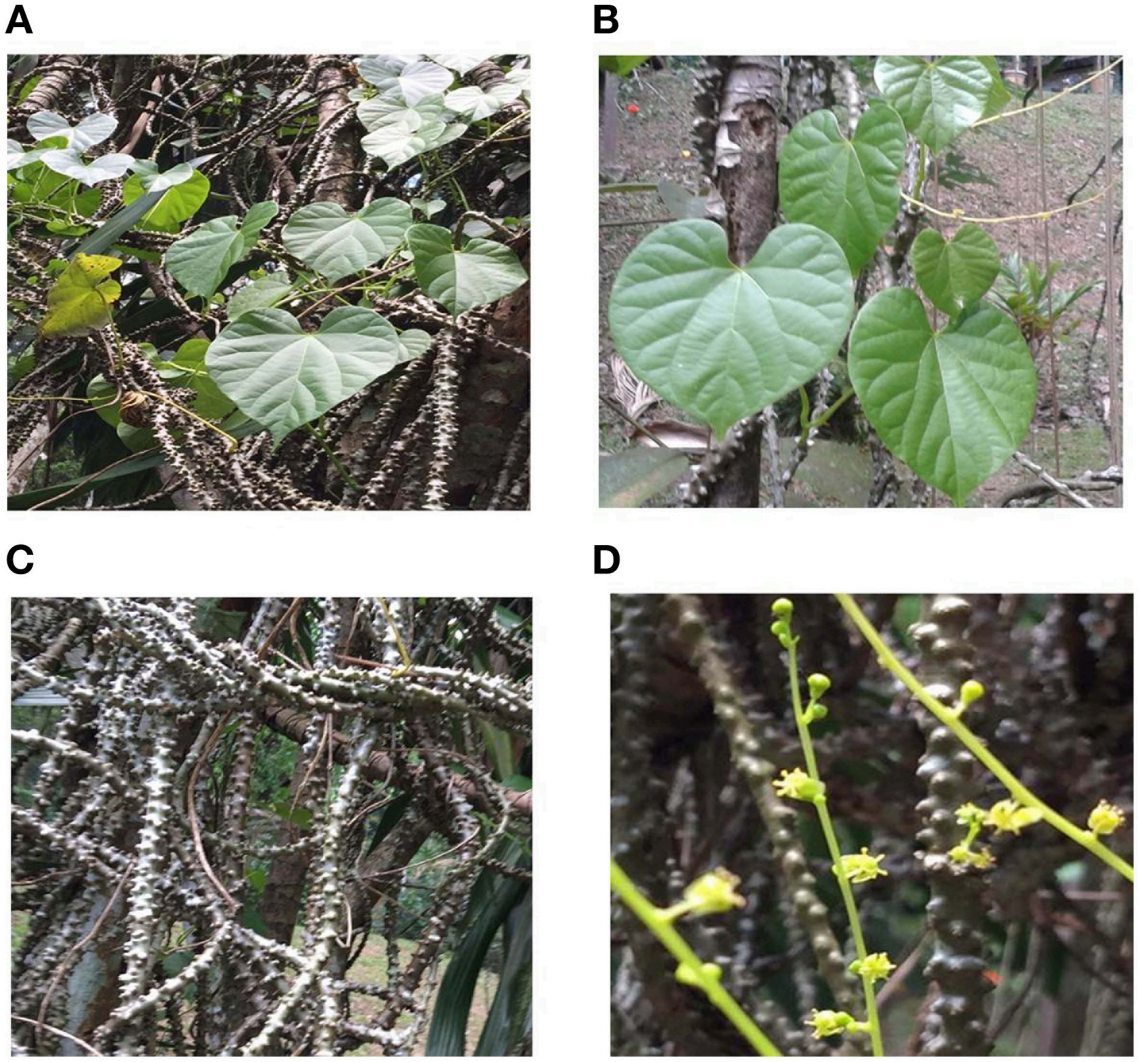
**(A)** whole plant of *Tinospora crispa* **(B)** leaves of *Tinospora crispa* **(C)** dried stem of *Tinospora crispa* **(D)** flowers of *Tinospora crispa*.

*T. crispa* is an herbaceous vine which extensively grows in tropical and subtropical regions of Southeast Asia (Pathak et al., 1995). The old stems of *T. crispa* are fleshy, with prominent blunt tubercles whereas younger stems are slightly fleshy, thin epidermis, membranous, brownish, and glabrous. The leaves are large, heart shaped 6–12 cm long and 7–12 cm wide. Petioles are glabrous and 5–15 cm long. Leaf blade is slightly fleshy, both surfaces glabrous and very delicate when dried (Figure 1). The herb contains two or three small and yellow or greenish yellow color flowers which are fascicled. Male inflorescences is very slender, 5–10 cm or longer. Male flower has six green and glabrous sepals in two whorls. Outer three are ovate (1 mm) while inner three are obovate. There are 3–6 yellow color petals and six stamens equivalent in length to petals. Female inflorescences are 2–6 cm long, mostly one flower per node. Female flower has sepals and petals as in male. The fruit is 7–8 mm in length.

The whole plant of *T. crispa* was obtained from Marang, Kuala Terengganu, Malaysia and a voucher specimen (No. UKMB 40178) was identified and deposited at the Herbarium of Universiti Kebangsaan Malaysia (UKM), Bangi, Malaysia. The collection of the plant sample did not involve endangered or protected species.

## Traditional uses

*T. crispa* is an ingredient in Thai folk remedies. Decoction from the stem of *T. crispa* has been used as an antipyretic, in the treatment of internal inflammations, decreasing thirst, enhancing hunger, cooling down body temperature, and for the maintenance of good health (Kongsaktrakoon et al., 1984; Dweck and Cavin, 2006). The cold infusion of the seed has been used to treat intoxication caused by drugs or alcohol. An infusion of its stem is drunk as vermifuge, a decoction of the stem is used to wash aching eyes and syphilitic sores, the crushed leaves are applied on wounds and made into dressing for itch. In Indonesia (Borneo) it has been used for the treatment of diabetes, hypertension, and backache (Dweck and Cavin, 2006). *T. crispa* has been used conventionally against a wide variety of health ailments by Yao communities of China. They used it to treat bruises, septicemia, fever, fracture, scabies, and other tropical ulcer-related disorders (Li et al., 2006). In Malaysia, *T. crispa* is used traditionally for numerous therapeutic purposes like diabetes, hypertension, stimulation of appetite, and protection from mosquito bites (Gimlette and Burkill, 1930). The infusion from the stems is used as a vermifuge. Personal communications with local traditional medicine practitioners highlighted its popular use as a general tonic. Moreover, it is used as an anti-parasitic agent in both humans and domestic animals (Noor et al., 1989). In Bangladesh, the juice of stem is used in the treatment of intestinal disorders, jaundice, rheumatism, body pain, paralysis, skin disease, and leprosy. The aqueous leaf extract is used to treat flatulence, dyspepsia, diarrhea, and rheumatism by traditional therapists in the Philippines. It is also used to prepare a poultice with coconut oil to treat arthritis. The traditional uses of *T. crispa* are summarized in Table 1 along with the parts used and methods of administration.

**Table 1:** **Traditional uses of ***Tinospora crispa*****.

**Country**	**Traditional name**	**Part used**	**Mode of use**	**Traditional use**	**References**
Thailand	Khruea khao ho-Boraphet	Stem Leaves Roots	Infusion Decoction Crushed leaves	Treatment of fever, cholera, diabetes, rheumatism, and snake-bites As a vermifuge As a wash for sore eyes and syphilitic sores. Treatment of wound itching and internal inflammation To reduce thirst and increase appetite	Kongsaktrakoon et al., 1984
		Seed	Cold infusion	Intoxication due to drugs or alcohol	Srithi et al., 2009
		stem	Decoction Chewing	Antipyretic, appetizer, bitter tonic, stomachic, eyes and ears problems, mucous congestion, intestinal parasites	Gimlette and Burkill, 1930
		stem	Decoction	Hemorrhoid	Chuakul et al., 2002
Indonesia	Antawali Brotowali	Stems	Infusion	Treatment of fever and malaria Treatment of stomachache and jaundice. To treat fevers caused by smallpox and cholera. Murut community traditionally uses *T. crispa* to treat diabetes, hypertension, lumbago, postpartum remedy and muscle pain	Roosita et al., 2008
Malaysia	akar patawali or akar seruntum	Whole plant	boiling	Kadazan-dusun community treats hypertension and malaria by drinking boiled plant	Ahmad and Ismail, 2003
Malaysia		Stem	Decoction	Tuberculosis Aqueous extract of *T. crispa* stems is taken orally to treat diabetes mellitus	Noor et al., 1989; Mohamad et al., 2011
Bangladesh	(Guloncho-ban) Golonchi	Stem	Juice obtained from macerated stems	Garo and Non-Garo traditional medicinal practitioners in Bangladesh use it for the prevention of intestinal disorders	Rahmatullah et al., 2011
		Vines	juice	A combination of *T. crispa* and *Saccharum officinarum* is used to treat fever	Islam et al., 2011
		Leaf stem	Juice	The leaf and stem juice is used to treat jaundice and rheumatism. To relieve body pain leaf and stem juice is massaged onto the affected area twice daily for 7 days	Rahmatullah et al., 2009
		Stem leaves	Pills Juice Boiling extract	Paralysis, abdominal pain, skin disease, leprosy	Kadir et al., 2014
China	Da ye ruan jin teng	Rattan		Yao communities of China use it for fracture, contusion, bitten by viper, carbuncle, furuncle, septicaemia, fever, scabies, and other tropical ulcer related disorders	Li et al., 2006
Cambodia	Banndol Pech	stem		Fever Rheumatism	Hout et al., 2006
Martinique island	(Lyann span Zeb kayenn)	Leaves Stem	Decoction(oral)	Anti diabetics	Longuefosse and Nossin, 1996
Philippine	Makabuhay	Leaf Stem	Aqueous extract	Treatment of flatulence, Indigestion, diarrhea, and rheumatism To treat arthritis when prepared as a poultice with coconut oil	Quisumbing, 1951

## Phytochemistry

*T. crispa* comprises of a diversity of secondary metabolites. A number of studies have been carried out on the constituents of *T. crispa*, and more than 65 compounds have been isolated and identified such as furanoditerpenes, lactones, steroids, flavonoids, lignans, and alkaloids (Table 2). Among these isolated compounds, clerodane-type furanoditerpenes are the characteristic compounds of *T. crispa*.

**Table 2:** **Chemical group, part of plant studied, and chemical constituents isolated from ***T. crispa*****.

**No**	**Chemical group**	**Part of plant**	**References**
**FLAVONE AND FLAVONE GLYCOSIDES**			
**1**	Apigenin	Stem	Lin, 2009
**2**	Diosmetin **(**Luteolin 4′-methyl ether)	Stem	Umi Kalsom and Noor, 1995
**3**	Genkwanin	Stem	Umi Kalsom and Noor, 1995
**4**	Luteolin 4′-methyl ether 7-glucoside	Stem	Umi Kalsom and Noor, 1995
**5**	Genkwanin 7-glucoside	Stem	Umi Kalsom and Noor, 1995
**6**	Luteolin 4′-methyl ether 3′-glucoside	Stem	Umi Kalsom and Noor, 1995
**TRITERPENE**			
**7**	Cycloeucalenol	Stem	Kongkathip et al., 2002
**8**	Cycloeucalenone	Stem	Kongkathip et al., 2002
**DITERPENE AND DITERPENE GLUCOSIDE**			
**9**	Tinocrispol A	Stem	Lam et al., 2012
**10**	Borapetol A	Whole plant	Fukuda et al., 1985; Chung, 2011
**11**	Borapetols B	Whole plant	Fukuda et al., 1986; Chung, 2011
**12**	2-*O*-lactoylborapetoside B	Stem	Lam et al., 2012
**13**	6′-*O*-lactoylborapetoside B	Stem	Lam et al., 2012
**14**	Borapetoside A	Stem	Martin et al., 1996; Chung, 2011
**15**	Borapetoside B	Stem	Martin et al., 1996; Chung, 2011
**16**	Borapetoside C	Stem	Martin et al., 1996; Chung, 2011
**17**	Borapetoside D	Stem	Martin et al., 1996; Chung, 2011
**18**	Borapetoside E	Stem	Martin et al., 1996; Chung, 2011
**19**	Borapetoside F	Stem	Martin et al., 1996; Chung, 2011
**20**	Borapetoside G	Stem	Choudhary et al., 2010b
**21**	Borapetoside H	Stem	Lam et al., 2012
**22**	Rumphioside A	Stem	Chung, 2011
**23**	Rumphioside B	Stem	Chung, 2011
**24**	Syringin	Stem	Cavin et al., 1998; Chung, 2011
**25**	Columbin	Stem	Lam et al., 2012
***CIS*****CLERODANE- TYPE FURANODITERPENOIDS**			
**26**	(3R,4R,5R,6S,8R,9S,10S,12S)-15,16-Epoxy-3,4-epoxy-6*-O-(β-D-*glucopyranosyl)-cleroda-3,13(16),14-trien-17,12-olid-18-oic acid methyl ester	Areial parts	Choudhary et al., 2010b
**27**	(1*R*,4*S*,5*R*,8*S*,9*R*,10*S*,12*S*)-15,16-Epoxy-4-*O*-(β-D-glucopyranosyl)-cleroda-2,13(16),14-triene-17(12),18(1)-diolide	Areial parts	Choudhary et al., 2010b
**28**	(2*R*,5*R*,6*R*,8*R*,9*S*,10*S*,12*S*)-15,16-Epoxy-2-hydroxy-6-*O*-(β-D-glucopyranosyl)-cleroda-3,13(16),14-trien-17,12-olid-18-oic acid methyl ester	Areial parts	Choudhary et al., 2010b
**29**	**(**5*R*,6*R*,8*S*,9*R*,10*R*,12*S*)-15,16-Epoxy-2-oxo-6-*O*-(β-D-glucopyranosyl)-cleroda-3,13(16),14-trien-17,12-olid-18-oic acid methyl ester	Areial parts	Choudhary et al., 2010b
**30**	(2*R*,5*R*,6*R*,8*S*,9*S*,10*S*,12*S*)-15,16-Epoxy-2-hydroxy-6-*O*-{β-D-glucopyranosyl-(1-6)*α-D*-xylopyranosyl}-cleroda-3,13(16),14-trien-17,12-olid-18-oic acid methyl ester	Areial parts	Choudhary et al., 2010b
**31**	Rumphiol E	Areial parts	Choudhary et al., 2010b
**32**	**(**5*R*,6*R*,8*S*,9*R*,10*S*,12*S*)-15,16-Epoxy-2-oxo-6-*O*-(β-D-glucopyranosyl)-cleroda-3,13(16),14-trien-17,12-olid-18-oic acid methyl ester	Areial parts	Choudhary et al., 2010b
**33**	(5R,6S,9S,10S,12S)-15,16-Epoxy-2-oxo-6-O-(β-D-glucopyranosyl)-cleroda-3,7,13(16),14-tetraen-17,12-olid-18-oic acid methyl ester	Areial parts	Choudhary et al., 2010b
**34**	(2R,5R,6S,9S,10S,12S)-15,16-Epoxy-2-hydroxy-6-O-(β-D-glucopyranosyl)-cleroda-3,7,13(16),14-tetraen-17,12-olid-18-oic acid methyl ester	Areial parts	Choudhary et al., 2010b
**ALKALOIDS**			
**35**	N-formylasimilobine 2-*O*-β-D-glucopyranoside	Stem	Choudhary et al., 2010a
**36**	N-formylasimilobine 2-*O*-β-D-glucopyranosyl-(1 → 2)-β-D-glucopyranoside	Stem	Fukuda et al., 1983; Choudhary et al., 2010a
**37**	Magnoflorine	Stem	Fukuda et al., 1983; Choudhary et al., 2010a; Yusoff et al., 2014
**38**	N-demethyl-N-formyldehydronornuciferine	Stem	Choudhary et al., 2010a
**39**	N-formylanonaine	Stem	Pachaly et al., 1992; Choudhary et al., 2010a; Yusoff et al., 2014
**40**	N-acetylanonaine	Stem	Pachaly et al., 1992; Lin, 2009
**41**	N-formylnornuciferine	Stem	Pachaly et al., 1992; Choudhary et al., 2010a; Yusoff et al., 2014
**42**	N-acetylnornuciferine	Stem	Pachaly et al., 1992; Chung, 2011;
**43**	Lysicamine	Stem	Sumimoto Chemicals Co Ltd, 1982
**44**	Tyramine	Stem	Praman et al., 2012
**45**	**Higenamine**	Stem	Praman et al., 2012
**46**	N-*cis*-feruloyltyramine	Stem	Chung, 2011
**47**	N-*trans-*feruloyltyramine	Stem	Choudhary et al., 2010a; Yusoff et al., 2014
**48**	Paprazine	Stem	Choudhary et al., 2010a
**49**	N-*trans*-caffeoyltyramine	Stem	Lin, 2009
**50**	4,13-dihydroxy-2,8,9-trimethoxydibenzo[a,g]quinolizinium	Stem	Yusoff et al., 2014
**51**	Columbamine	Stem	Yusoff et al., 2014
**52**	Dihydrodiscretamin	Stem	Yusoff et al., 2014
**53**	Palmatine	Stem	Sumimoto Chemicals Co Ltd, 1982
**54**	Jatrorrhizine	Stem	Sumimoto Chemicals Co Ltd, 1982
**55**	Berberine	Stem	Bisset and Nwaiwu, 1983
**56**	Salsolinol	Stem	Praman et al., 2012
**57**	(−)-Litcubinine	Stem	Praman et al., 2012
**LIGNAN**			
**58**	Secoisolariciresinol	Stem	Chung, 2011
**59**	Syringaresinol	Stem	Chung, 2011
**60**	Adenosine	Stem	Praman et al., 2012
**61**	Uridine	Stem	Praman et al., 2012
**62**	Adenine	Stem	Praman et al., 2012
**STEROL**			
**63**	β-sitosterol	Stem	Lin, 2009
**64**	Stigmasterol	Stem	Lin, 2009
**65**	Makisterone C	Stem	Lin, 2009

### Flavonoids

**Figure 2:**
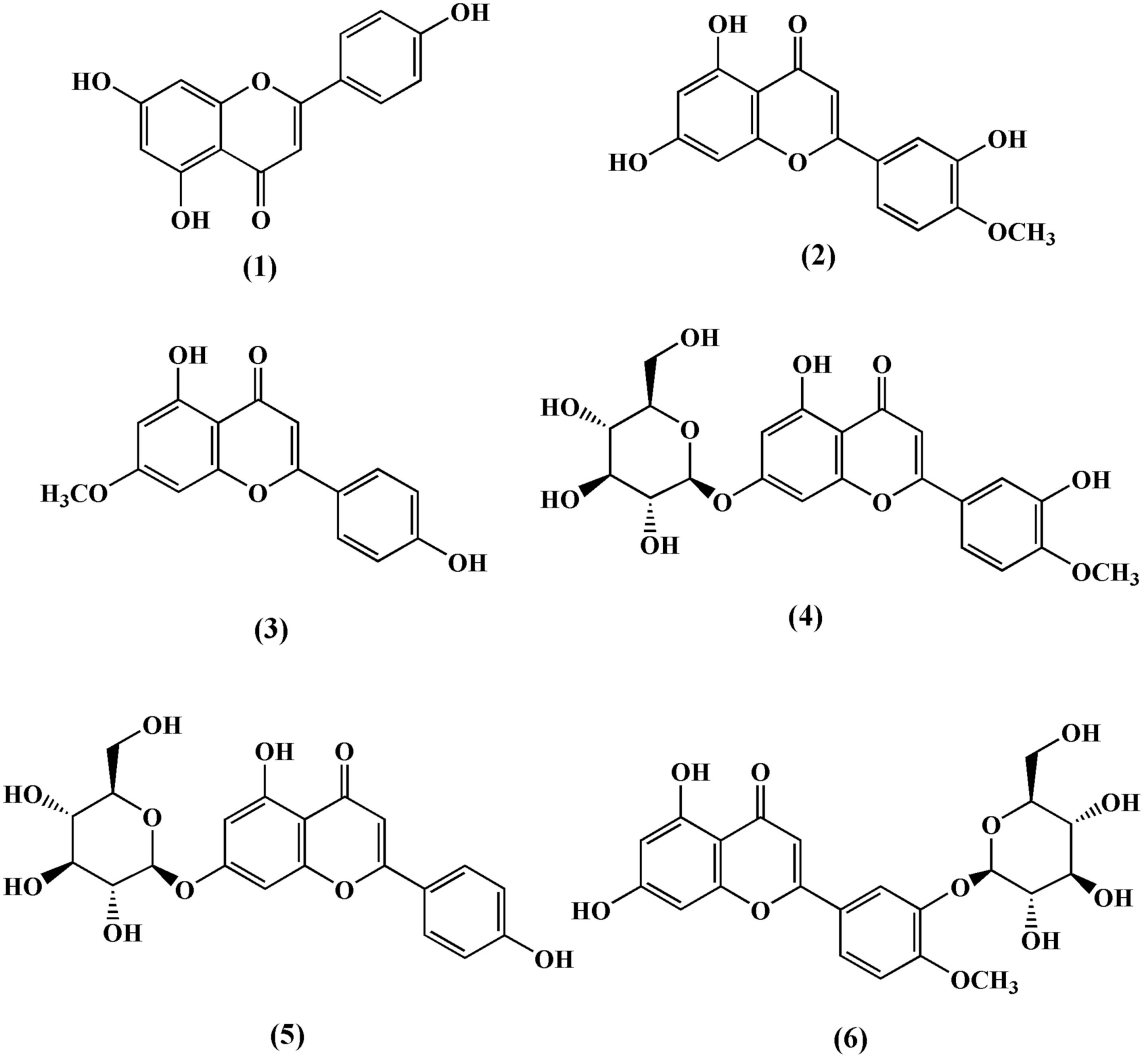
**Chemical structures of flavones and flavone glycosides isolated from ***Tinospora crispa*****.

Till date, three flavones and three flavone glycosides have been identified from the stem of *T. crispa*, namely, apeginin **(1)** (Lin, 2009), diosmetin **(2)**, genkwanin **(3)**, luteolin 4′-methyl ether 7-glucoside **(4)**, genkwanin 7-glucoside **(5)**, and luteolin 4′-methyl ether 3′-glucoside **(6)** (Umi Kalsom and Noor, 1995; Figure 2).

### Terpenoids

**Figure 3:**
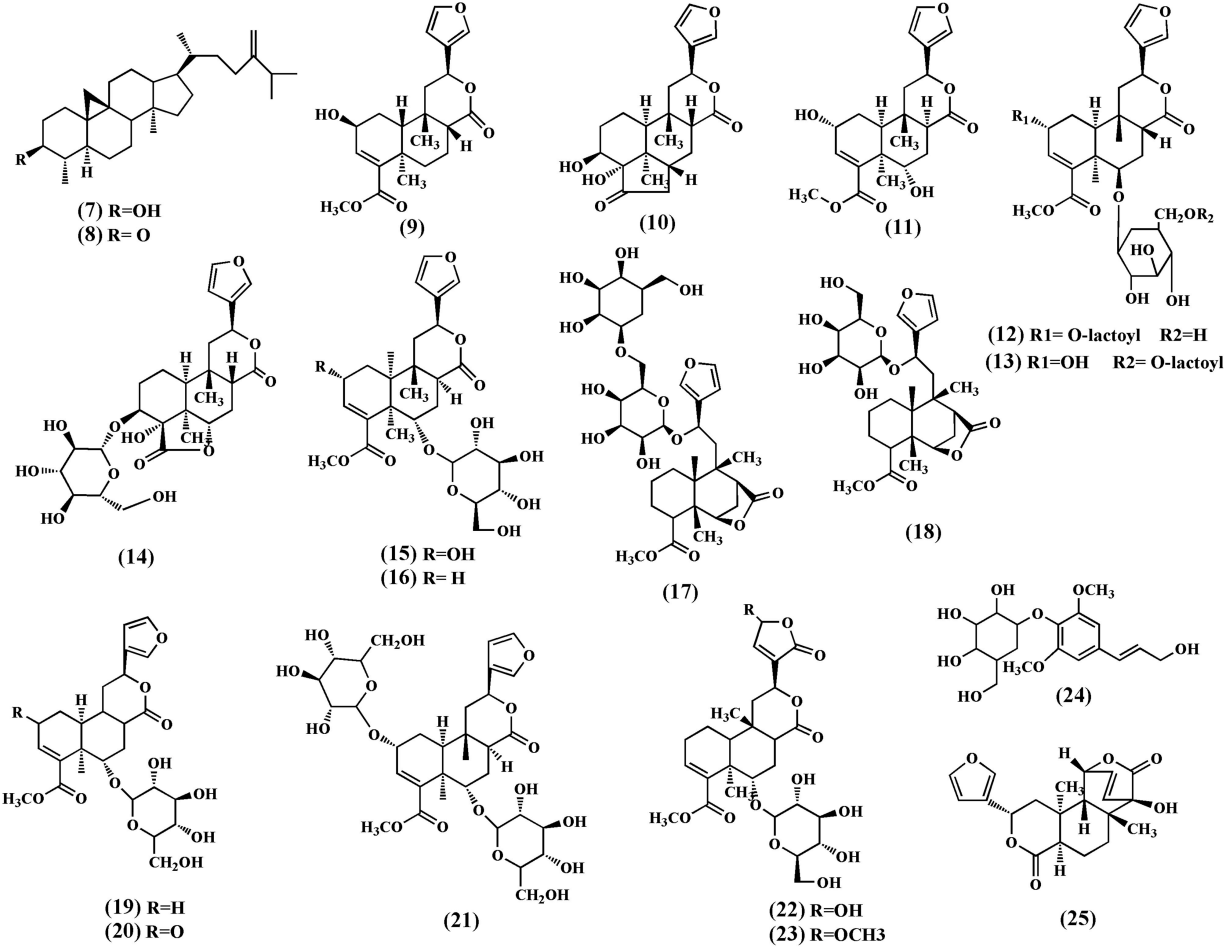
**Chemical structures of terpenoids obtained from ***Tinospora crispa*****.

A number of terpenoids (**7–33**), classified as triterpenoids (**7–8**), and diterpenoids (**9–34**), have been isolated from different parts of *T. crispa*. The triterpenoids, cycloeucalenol **(7)** and cycloeucalenone **(8)** were also isolated from the stem (Kongkathip et al., 2002). Diterpenoids and their glycosides are the main terpenoids in *T. crispa* and the most common are the clerodane-type furanoditerpenoids. Diterpenoids, tinocrispol A **(9)** (Lam et al., 2012), borapetol A **(10)**, borapetols B **(11)**, were isolated from the ethanol extract of *T. crispa* vines (Fukuda et al., 1985, 1986; Chung, 2011; Figure 3).

Diterpenoid glycosides, 2-*O*-lactoylborapetoside B **(12)**, 6′-*O*-lactoylborapetoside B **(13)**, borapetoside A **(14)**, borapetoside B **(15)**, borapetoside C **(16)**, borapetoside D **(17)**, borapetoside E **(18)**, borapetoside F **(19)** (Martin et al., 1996), borapetoside G **(20)**, borapetoside H **(21)**, rumphioside A **(22)**, rumphioside B **(23)**, rumphioside C, rumphioside F, rumphioside I, syringin **(24)**, columbin **(25)**, tinocrisposide A, tinocrisposide B, tinocrisposide C, and tinocrisposide D were isolated from the methanol extract of *T. crispa* (Chung, 2011; Lam et al., 2012; Figure 3).

**Figure 4:**
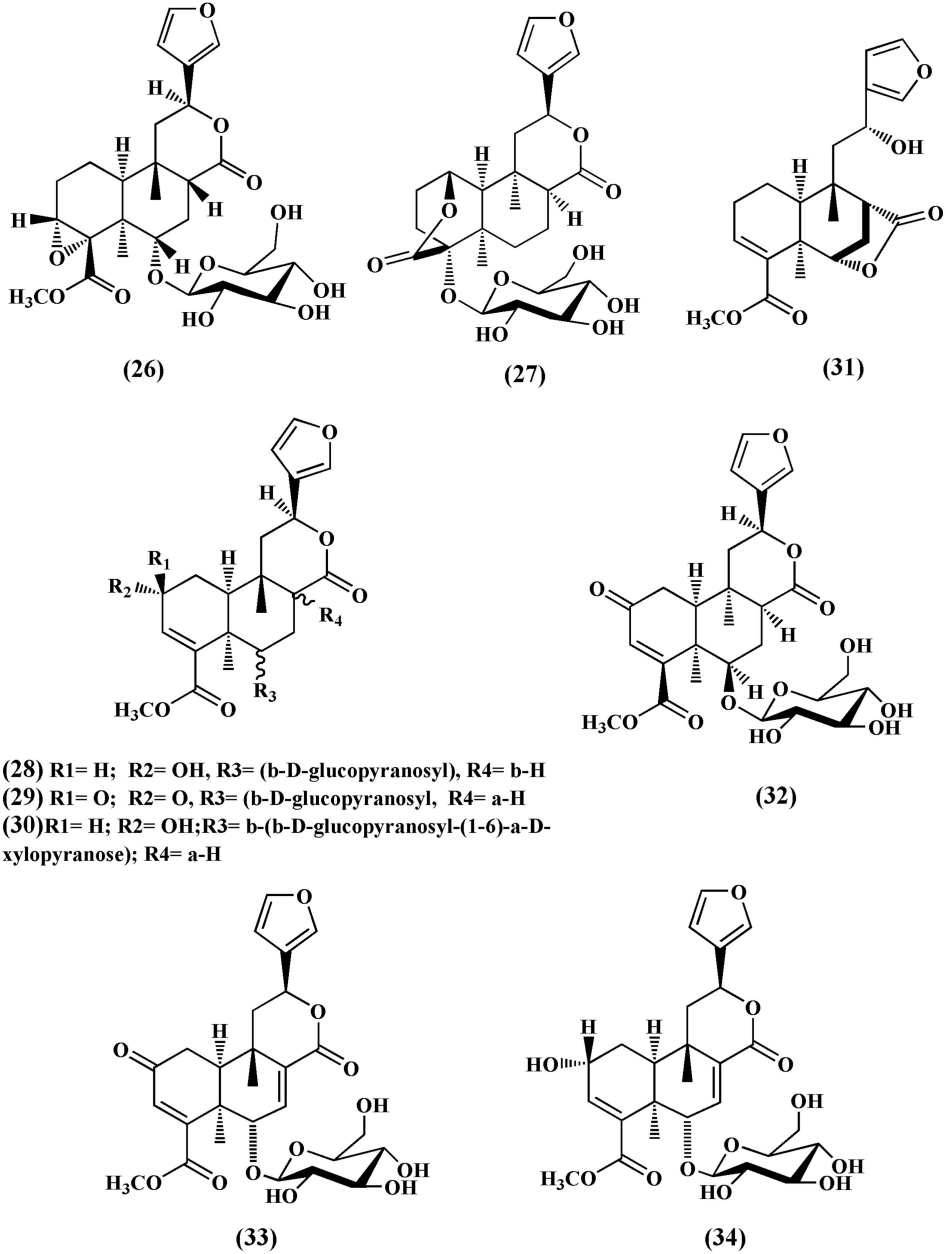
*****Cis***clerodane-type furanoditerpenoids isolated from ***Tinospora crispa*****.

Choudhary et al. (2010b) also isolated nine new *cis* clerodane-type furanoditerpenoids, from aerial parts of *T. crispa*, viz. (3R,4R,5R,6S,8R,9S,10S,12S)-15,16-epoxy-3,4-epoxy-6*-O-(*β*-D-* glucopyranosyl)-cleroda-3,13(16),14-trien-17,12-olid-18-oic acid methyl ester **(26)**, (1*R*,4*S*,5*R*,8*S*,9*R*,10*S*,12*S*)-15,16-epoxy-4-*O* -(β-D-glucopyranosyl) -cleroda-2,13(16),14-triene-17(12),18(1)-diolide **(27)**, (2*R*,5*R*,6*R*,8*R*,9*S*,10*S*,12*S*)-15,16-epoxy-2 -hydroxy-6-*O*-(β-D-glucopyranosyl)-cleroda-3,13(16),14-trien-17,12-olid-18-oic acid methyl ester **(28)**, **(5)***R*,6*R*, 8*S*,9*R*,10*R*,12*S*)-15,16-epoxy-2-oxo-6-*O*-(β-D-glucopyranosyl)-cleroda-3,13(16),14-trien-17,12-olid-18-oic acid methyl ester **(29)**, (2*R*,5*R*,6*R*,8*S*,9*S*,10*S*,12*S*)-15,16-epoxy-2-hydroxy-6-*O*-{β-D-glucopyranosyl-(1-6)α*-D*-xylopyranosyl}-cleroda-3,13(16),14- trien-17,12-olid-18-oic acid methyl ester **(30)**, rumphiol E **(31)**, **(**5*R*,6*R*,8*S*,9*R*,10*S*,12*S*)-15,16-epoxy-2-oxo-6-*O*-(β-D-glucopyranosyl)-cleroda-3,13(16),14-trien-17,12-olid-18-oic acid methyl ester **(32)**, (5R,6S,9S,10S,12S)-15,16-epoxy-2-oxo-6-O-(β-D-glucopyranosyl)-cleroda-3,7,13(16),14-tetraen-17,12-olid-18-oic acid methyl ester **(33)**, and (2R,5R,6S,9S,10S,12S)-15,16-epoxy-2-hydroxy-6-O-(β-D-glucopyranosyl)-cleroda-3,7,13(16),14-tetraen-17,12-olid-18-oic acid methyl ester **(34)** (Figure 4).

### Alkaloids

**Figure 5:**
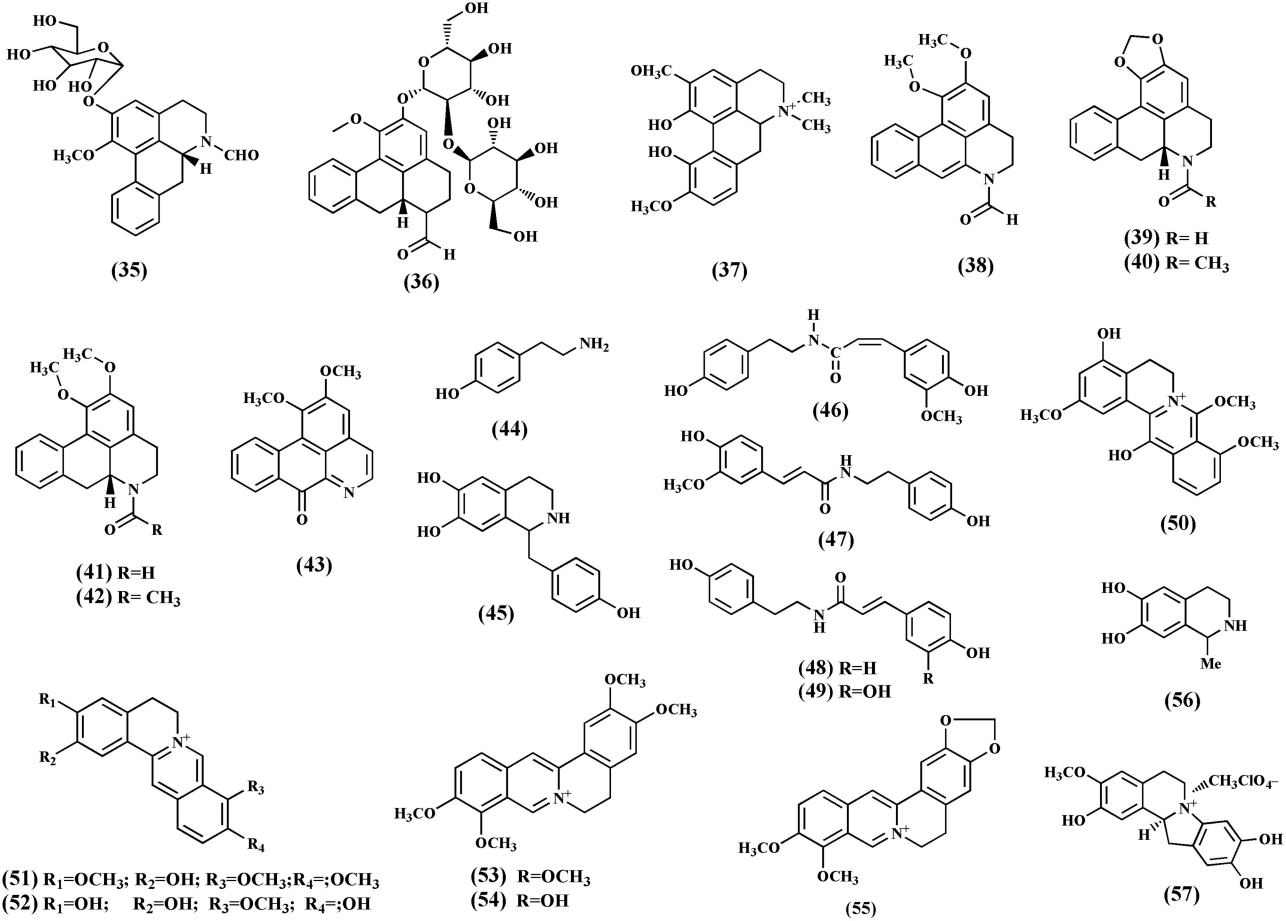
**Chemical structures of alkaloids present in ***Tinospora crispa*****.

Alkaloids are important secondary metabolites from the plant. To date, 21 quaternary alkaloids have been isolated (**35-57**) and classified into protoberberine, furonoquinolone, and aporphine alkaloids (Figure 5). The most common alkaloids found in *T. crispa* are aporphines. These include *N*-formylasimilobine 2-*O*-β-D-glucopyranoside **(35)**, *N*-formylasimilobine 2-*O*-β- D-glucopyranosyl-(1 → 2)-β-D-glucopyranoside (tinoscorside A) **(36)**, magnoflorine **(37)**, *N*-demethyl-*N*-formyldehydronornuciferine **(38)** (Fukuda et al., 1983; Choudhary et al., 2010a), *N*-formylanonaine **(39)**, *N*-acetylanonaine **(40)**, *N*-formylnornuciferine **(41)**, *N*-acetylnornuciferine **(42)** (Pachaly et al., 1992; Na et al., 2005), and lysicamine **(43)** (Sumimoto Chemicals Co Ltd, 1982). The furquinolone alkaloids isolated from *T. crispa* comprise tyramine **(44)**, higenamine **(45)** (Praman et al., 2012), *N-cis-*feruloyltyramine **(46)**, *N*-*trans-*feruloyltyramine **(47)**, paprazine **(48)**, and *N*-*trans-*caffeoyltyramine **(49)** (Naomichi et al., 1983; Chung, 2011). The protoberberine alkaloids include 4,13-dihydroxy-2,8,9-trimethoxydibenzo[a,g]quinolizinium **(50)**, columbamine **(51)**, dihydrodiscretamin **(52)** (Yusoff et al., 2014), palmatine **(53)**, jatrorrhizine **(54)** (Sumimoto Chemicals Co Ltd, 1982), and berberine **(55)** (Bisset and Nwaiwu, 1983). Salsolinol **(56)** (a tetrahydroisoquinoline) and (−)-Litcubinine **(57)** (a dibenzopyrrocoline type alkaloid) were identified from n-butanol fraction of *T. crispa* stem (Praman et al., 2012).

### Lignans, nucleosides, and sterols

**Figure 6:**
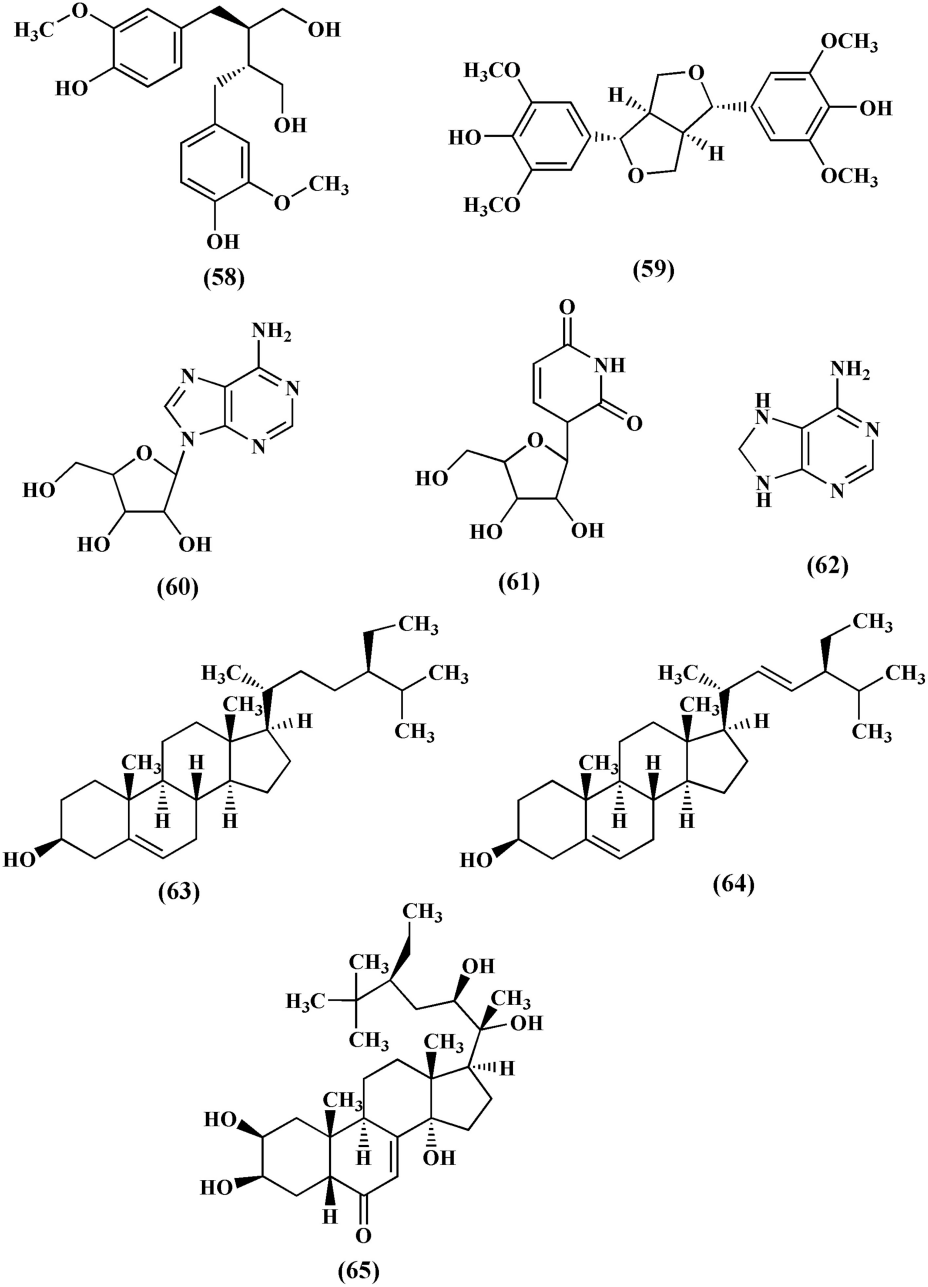
**Chemical structures of lignans, nucleosides and sterols found in ***Tinospora crispa*****.

Lignans are group of compounds that arise from the shikimic acid pathway. Secoisolariciresinol **(58)** and syringaresinol **(59)** are lignans isolated from the methanol extract of *T. crispa* (Chung, 2011). Adenosine **(60)**, uridine **(61)**, and adenine **(62)** are the nucleosides isolated from the *n*-butanol fraction of *T. crispa* stem (Praman et al., 2012; Figure 6). Sterols like β-sitosterol **(63)**, stigmasterol **(64)** and makisterone C **(65)** have also been isolated from *T. crispa* (Lin, 2009; Figure 6).

## Pharmacological properties

### Anti-inflammatory and immunomodulatory activities

The crude ethanol extract of *T. crispa* together with other fractions were investigated for potential anti-inflammatory activity by evaluating their effect on expression of intracellular cytokine in LPS stimulated murine macrophage cell line RAW264.7 (murine macrophages from blood). *T. crispa* crude extract and its isolated fractions stimulated RAW264.7 proliferation in a dose dependent way. *T. crispa* crude extract at a dose of 25–800 μg/mL expressively increased RAW264.7 proliferation (Table 3). The ethanol extract of *T. crispa* and its fractions also improved intracellular expressions of cytokine, INF-γ, IL-6, and IL-8. Among all the fractions tested, ethyl acetate fraction was the most active which exhibited significant (*P* ≤ 0.05) increase in the intracellular expressions of cytokines in RAW264.7 macrophages (Abood et al., 2014). This suggested that the compounds which exhibited immunomodulatory activity were soluble in ethyl acetate. Four active compounds, i.e., cordioside, quercetin, paullinic acid, and boldine were identified by LC-MS analysis of the ethyl acetate fraction. However, chromatography of ethyl acetate fraction to further isolate and characterize the active constituents was not performed. Besides, the active constituents should also be studied at a molecular level to explore their mechanisms of action and role as immunomodulators.

**Table 3:** **Summary of the Pharmacological activities of ***Tinospora crispa*****.

**Pharmacological activity**	**Tested substance**	**Model used**	**Tested Dose**	**Results**	**Reference**
Anti-inflammatory activity	Aqueous, Methanol stem extract	TNF-α induced inflammation in Human umbilical vein endothelial cells	HUVECs were incubated at concentrations: 100- 200-400-600 μg/mL	Both extracts showed inhibition of signaling molecules ICAM-1, VCAM-1, MCP-1, M-CSF, while the secretion of NO was increased	Kamarazaman et al., 2012a
	Methanol extract	Carrageenan induced inflammation (edema) in Sprague-dawley rats	30, 100, and 300 mg/kg intraperitonial	The methanol extract significantly inhibited the development of edema	Hipol et al., 2012
Immunomodulatory effect	Ethanol extract And isolated fractions	Detemination of intracellular cytokine in LPS stimulated murine macrophage cell line RAW264.7	25–1000 μg/mL	*T. crispa* crude extract and its isolated fraction stimulate RAW264.7 cell viability and intracellular expressions of cytokine,INF-γ, IL-6, and IL-8	Abood et al., 2014
Cytotoxic activity	water, methanol and chloroform whole plant extract	MCF-7, MDA-MB-231, HeLa, and 3T3 fibroblast cells	10–100 μg/mL of each extract	All extracts showed dose-dependent antiproliferative activity	Ibahim et al., 2011
	Methanol stem Extract	HL-60 leukemic cells, HepG2 hepatoma cells and Hep3B hepatoma cells, containing virus	Cells were incubated with 0.03- 1 mg/mL concentarion range for 72 hrs	Inhibition was observed by methanol stem extract with IC_50_ HL-60; 0.12 mg/mL HepG2; 1.03 mg/mL Hep3B; 0.16 mg/mL	Sinchaikul et al., 2007
	aqueous crude extract of *T. crispa* stem	MTT assays using human cancer cell lines; MCF-7 HeLa Caov-3 HepG2	Cells were incubated with 10-200 μg/mL concentarion range for 72 h	MCF-7: 107 μg/mL, HeLa; 165 μg/mL, Caov-3;100 μg/mL HepG2;: 165 μg/mL	Amom et al., 2008
Antioxidant	water, methanol and chloroform whole plant extract	DPPH free radical scavenging assay	10–100 μg/mL	Methanol extract significantly increased percentage radical scavenging activity with IC_50_ value 12 μg/mL and percentage radical activity increased to 100% which was similar to vitamin	Ibahim et al., 2011
	Methanol extract of stem	DPPH free radical scavenging activity	0.1–0.5 mg/kg	The methanolic extract showed inhibition of DPPH with IC_50_ value 0.118 mg/mL	Zulkefli et al., 2013
	Methanol extract of stem	Metal chelating assay	0.0625–1 mg/mL	The extract showed inhibition of metal chelating	Zulkefli et al., 2013
	Methanol extract of stem	Reducing Power Assay	0.0625–1 mg/mL	The extract showed antioxidant effect by reducing ferric ion (Fe3+) to ferrous ion (Fe2+)	Zulkefli et al., 2013
Antinociceptive Activity	Ethanol extract of stem	Hot plate method and Acid acetic-induced writhes in male Balb C mice	30, 100, and 300 mg/kg intraperitonial	The extract exhibited antinoceceptive response, significant reduction in acetic acid induced writhing response	Sulaiman et al., 2008
Antimalarial activity	Methanol extract	chloroquine-sensitive malaria parasite (Plasmodium berghei ANKA) infected ICR mice model	20, 100, and 200 mg/kg intraperitonial	The extract exhibited antimalarial activity in dose-dependent manner	Niljan et al., 2014
	methanol extract	antiplasmodial activity method based on growth of *Plasmodium falciparum*	0.1–2.5 mg/mL	100% inhibition of parasite growth observed at 72 h at 2.5 mg/mL	Najib Nik a Rahman et al., 1999
	methanol extract	Adult female ddy mice model infected intraperitoneally with parasitised red blood cells. (Plasmodium berghei ANKA)	5 mg/kg Intra peritonial	The extract showed inhibitory effect on parasite growth	Najib Nik a Rahman et al., 1999
	Aqueous extract	Antiplasmodial activity method based on the measurement of growth of parasites. Species: chloroquine-sensitive strain Plasmodium falciparum		Plant extract IC_50_ 25 μg/mL	Bertani et al., 2005
	Aqueous extract	Determine effect on intraerythrocytic cycle and intrahepatic cycle in Plasmodium yoelii yoelii 17X strain infected swiss female mice model	110 mg/kg oral	The extract presented good antimalarial activity, inhibited more than 50% of the parasite development at dose of 110 mg/kg	Bertani et al., 2005
Hypoglycaemic effect	Aqueous extract	alloxan-diabetic male wistar albino rats model	4 g/L extract dissolved in drinking water	The extract treated diabetic animals exhibited lower fasting blood glucose levels and higher serum insulin levels	Noor et al., 1989
	95% ethanolic extract	Normoglycemic and alloxan-diabetic male Sprague-dawley rats model	125, 250, and 500 mg/kg oral administration	The blood sugar level of diabetic rats decreased after receiving the extract	Anulukanapakorn et al., 2012
	*n*-butanol fraction, Ether fraction, Aqueous fraction	Normoglycemic and alloxan-diabetic male Sprague-dawley rats model	50, 150, and 450 mg/kg Oral administration	*n*-butanol fraction and aqueous fraction of *T. crispa* exhibited significant hypoglycemic effect in alloxan-diabetic rats	Anulukanapakorn et al., 2012
	Two diterpenoids borapetosides A and C isolated from ethanolic extract of vines	Induced Type 1 and Type2 diabetic induced ICR mice. Type 1 induced by ip injection of streptozotocin and type 2 induced by fat-rich chow and 20% fructose-sweetened water	5 mg/kg Ip	The borapetosides A-C showed lowering of plasma glucose levels in normal and streptozotocin-induced type 1 diabetic mice. Borapetoside C increased glucose utilization in peripheral tissues and reduced hepatic gluconeogenesis	Lam et al., 2012
	Borapetol B	Determine blood glucose and plasma insulin in normoglycemic Wistar and type 2 diabetic Goto-Kakizaki rats by an oral glucose tolerance test	10 μg/100 g body weight	Blood glucose level significantly decreased and insulin level increased in both borapetol B treated normoglycemic Wistar and type 2 diabetic Goto-Kakizaki rats as compared to the placebo group	Lokman et al., 2013
	Borapetol B	Insulin secretion using isolated pancreatic islets by batch incubation and perifusion	0.1, 1, and 10 μg/mL	Borapetol B increased secretion of insulin from isolated islets in a dose-dependent manner	Lokman et al., 2013
	4,13-dihydroxy-2,8,9-trimethoxydibenzo[a,g]quinolizin	*Acetylcholinesterase (AChE) Inhibitory Activity* by using Ellman's colorimetric method	62.5–1000 μg/mL	Weak inhibition IC_50_:517.6 ± 5.3 μM	
	dihydrodiscretamine		62.5–1000 μg/mL	Moderate inhibition IC_50_: 276.1 ± 1.8 μM	
	columbamine		62.5–1000 μg/mL	strongest AChE inhibition IC_50_: 48.1 ± 1.3μM	
	magnoflorine		62.5–1000 μg/mL	*No inhibition*	
	*N*-formylannonaine		62.5–1000 μg/mL	Moderate inhibition IC_50_: 415.3 ± 2.7 μM	
	*N*-formylnornuciferine		62.5–1000 μg/mL	Weak inhibition IC_50_: 564.6 ± 2.1 μM	
	*N*-*trans*-feruloyltyramine		62.5–1000 μg/mL	*No inhibition*	
Antifilarial effects	The aqueous extract of dried stems	Microfilaricidal activity based upon microfilarial motility Worms: subperiodic *Brugia malayi*		The aqueous extract of *T. crispa* was found to exhibit microfilaricidal activity which was investigated based upon direct observation of the microfilarial	Zaridah et al., 2001
Cardiovascular activity	n-butanol stem extract	Determine effect on blood pressure and heart rate in normal and reserpinized (5 mg/kg) female wistar rats model	1–100 mg/kg, i.v	The extract modified the actions of the human cardiovascular system. In reserpinized rats, the *T. crispa* extract had a dual effect: reduction in hypotensive activity, followed by a small increase in blood pressure while in normal rat a small decrease in beginning followed by an increase in heart rate	Praman et al., 2011
	Active components isolated, higenamine, tyramine, salsolinol uridine, and adenosine	Determine effect on blood pressure and heart rate in normal and reserpinized (5 mg/kg) female wistar rats model	Salsinol 0.1–10 mg/kg Higenamine (0.001–0.3 mg/kg) Tyramine (0.003–1 mg/kg) Adenosine (0.003–0.3 mg/kg) Uridine (0.1–100 mg/kg) IV	Salsolinol, Adenosine decreased mean arterial blood pressure, and heart rate while Uridine increased mean arterial blood pressure and decreased heart rate. Higenamine decreased mean arterial blood pressure and increased heart rate. Tyramine increased mean arterial blood pressure and heart rate in normal rats	Praman et al., 2013

The activity of the aqueous extract of *T. crispa* stem on nitric oxide (NO) production in LPS stimulated peritoneal macrophages was studied by Yokozawa et al. (2000) using Griess reagent method. The aqueous extract exhibited a dose-dependent (from 5 to 250 μg/mL) inhibition of NO production. Bioassay-guided isolation of the aqueous extract showed that N-trans-feruloyltyramine was the active constituent responsible for the inhibition of NO and this inhibition was linked with reduced levels of inducible NO synthase (iNOS) expression. (Yokozawa et al., 2000, 2001).

The secretion of macrophage colony stimulating factor (M-CSF), vascular cell adhesion molecule (VCAM-1), and intracellular cell adhesion molecule (ICAM-1) in TNF-α stimulated human umbilical vein endothelial cells (HUVECs) was reduced by the aqueous and methanol extracts of *T. crispa* stem (Kamarazaman et al., 2012a). These adhesion molecules (ICAM-1, VCAM-1) and an inflammatory signaling molecule (M-CSF) were reported to be up-regulated during an immune response. The recruitment of leukocytes was dependent on ICAM-1 and VCAM-1 (Kamarazaman et al., 2012a).

The effect of the aqueous and methanol extracts of *T. crispa* on cell-mediated immune response was evaluated by foot pad reaction. The development of edema was significantly inhibited by the aqueous extract at doses of 50, 100, and 150 mg/kg and the results were equivalent to ibuprofen (Hipol et al., 2012). The early and late phases of carrageenan-induced inflammation were affected by the extract. Furthermore, oral administration of 50% methanol extract of stem, at a dose of 10 mg/kg also inhibited the carrageenan-induced edema in rats as compared to control group (Higashino et al., 1992). However, these results differ from those reported by Aher and Kumar Wahi (2010) for the other plant of the same genus (*T. cardifolia*). According to Aher and Kumar Wahi (2010), the methanol extract of *T. cardifolia* increased the foot pad thickness in rats, at a dose of 100 mg/kg.

Apart from the above-mentioned reports, there is deficiency of data to offer proofs for anti-inflammatory activity. Actually, adequate studies have not been performed on the inflammatory cells, proinflammatory cytokines, and proinflammatory enzymes (PLA_2_, COX and LOX, PGE_2_). The assessment of the effect of pure compounds and fractions on the activity and gene expression of enzymes and cytokines involved in inflammation might be beneficial. Likewise, reactive oxygen species including superoxide anion, hydroxyl radical, hydrogen peroxide, and singlet oxygen play a vital part in pathogenesis of inflammation. It would be exciting to assess the effect on these reactive oxygen species. Taking together these results, it is quite premature to conclude regarding the anti-inflammatory activity of *T. crispa*.

### Anticholinesterase activity

The hydrolysis of acetylcholine to choline is catalyzed by an enzyme acetylcholinesterase (AChE). The hydrolysis of acetylcholine results in the end of nerve impulse transmission at the cholinergic synapses. The quarternary alkaloids, (4,13-dihydroxy-2,8,9-trimethoxydibenzo quinolizinium, magnoflorine, columbamine, *N*-formylannonaine, dihydrodiscretamine, *N*-formylnornuciferine, and *N*-*trans*-feruloyltyramine) isolated from *T. crispa* were investigated as inhibitors of AChE by using Ellman's colorimetric method. The isolated compounds showed different activity profiles. Among all the compounds, columbamine displayed the strongest AChE inhibitory activity with an IC_50_ value of 48.1 μM which was comparable to that of physostigmine (IC_50_ 31.4 μM; Yusoff et al., 2014). A number of alkaloids isolated from medicinal plants have been reported for their AChE inhibitory activity. Alkaloids isolated from *T. crispa* should be evaluated for their AChE inhibitory activity. The AChE inhibition has therapeutic potential for treatment of parkinson's and alzheimer's diseases, senile dementia, ataxia, and myasthenia gravis. Nevertheless, the results presented in above-mentioned study are not sufficient to draw a meaningful conclusion. Hence, more cutting-edge and mechanistic studies are needed to better understand the anticholinesterase activity.

### Antibacterial and antifilarial activities

Aqueous, ethanol and chloroform extracts of *T. crispa* were evaluated for their antimicrobial activity against some gram-positive (*Bacillus cereus, Staphylococcus aureus, Listeria monoctogens, Streptococcus pneumonia, and Clostridium diphtheria*) and gram-negative bacteria (*Shigella flexneri, Salmonella typhi, Proteus vulgaris, Escherichia coli, and Klebsiella pneumonia*). The activity of *L. monoctogens and P. vulgaris* was slightly inhibited by all the extracts. The ethanol extract was effective against *S. pneumonia, S. aureus, S. flexneri, and C. diphtheria* while chloroform extract inhibited the activities of *S. flexneri, C. diphtheria, and S. pneumonia*. However, *E. coli, B. cereus*, and *S. typhi* remained unaffected by all the extracts (Zakaria et al., 2006). The above-presented results do not coincide with the findings of Md and Mohammad (2011) and Chittur and Gunjan (2012). According to Md and Mohammad (2011), the chloroform extract of *T. crispa* inhibited *E. coli, B. cereus*, and *S. typhi* with zone of inhibitions of 7, 8, and 9 mm, respectively. On the other hand, Chittur and Gunjan (2012) reported that at a dose of 50 μg/mL, the aqueous and ethanol extracts of *T. crispa* inhibited *E.coli with* zone of inhibition of 2.6 and 3.6 mm, respectively. As no dose-dependent study was performed, it is difficult to evaluate the minimum inhibitory concentration (MIC) and the minimum bactericidal concentration (MBC). The antibacterial studies carried out by Al-alusi et al. (2010) have shown worth mentioning antibacterial activity of *T. crispa* extracts against the methicillin-resistant *S. aureus* (MRSA) as compared to the control (vancomycin). The traditional use of *T. crispa* in the treatment of cholera and syphilitic sores could be validated by investigating its inhibitory effect on the activity of *Vibrio cholera* and *Treponema pallidum*. The antibacterial activity of *T. crispa* needs to be extensively studied and the mechanism involved in the antibacterial activity should also be further explored.

The aqueous extract of dried stems of *T. crispa*, investigated for *in vitro* antifilarial effects, showed moderate activity against the adult worms of sub periodic *Brugia malayi* whereby the value of relative movability values were used as a measure of the antifilarial activity (Zaridah et al., 2001). The aqueous extract of *T. crispa* exhibited microfilaricidal activity which was investigated based on direct observation of the microfilarial motility (Merawin et al., 2010). The bioactive compounds contributing to the antifilarial activity should be isolated and further studies need to be carried to study their mechanisms of action.

### Antioxidant activity

On the basis of DPPH, FRAP, and TBA tests, the aqueous crude extract of *T. crispa* stem was found to display high antioxidant activity and its antioxidative potency was equivalent to the previously established antioxidants like BHT and vitamin C (Amom et al., 2008; Zulkhairi et al., 2009). The antioxidant assay performed by Froemming (2011) exhibited that the methanol extract of *T. crispa* displayed the highest antioxidant activity which was determined by measuring total flavonoid content, total phenolic content, and DPPH free radical scavenging activity. The antioxidant activity could be attributed to the phenolic compounds present in *T. crispa* such as flavonoids that act as free radical scavengers. Cavin et al. (1998) isolated vanillin, syringin, *N*-formylannonain, *N-*formylnornuciferin, borapetosides B, C, and F, *N cis*- feruloyltyramine, *N-trans*-feruloyltyramine, and secoisolariciresinol from the dichloromethane extract of *T. crispa*. Antioxidant and free-radical scavenging potency of *N-cis*-feruloyltyramine, *N-trans*- feruloyltyramine, and secoisolariciresinol were higher than the synthetic antioxidant butylhydroxytoluene (BHT). The antioxidant activity could be of therapeutic importance in preventing oxidative stress involved in the development of several diseases including cardiovascular and neurological disorders.

### Atherosclerosis inhibitory activity

Amom et al. (2011) discovered that the aqueous extract from *T. crispa* stem administered to hypercholesterolemic rabbits delayed the development of atherosclerosis by suppressing the levels of total cholesterol, triglycerides and low density lipoproteins. In contrast, the level of high density lipoproteins was found to be significantly increased. Furthermore, they also demonstrated that the aqueous and methanol extracts of *T. crispa* decreased the malondialdehyde level in a dose-dependent manner by increasing the activity of antioxidant enzymes (catalase, superoxide dismutase, and glutathione peroxidase) in H_2_O_2_ induced HUVECs (Kamarazaman et al., 2012b). These antioxidant enzymes have been reported to inhibit the reactive oxygen species that actively oxidize the LDL in blood and result in the development of atherosclerosis. The above-mentioned studies do not provide comprehensive and sufficient information. The range of the tested doses was very narrow and no information about the positive control, EC_50_ and IC_50_ have been provided. Hence, it is too early to conclude about the antiatherosclerosis activity of *T. crispa*. However, these findings showed that *T. crispa* possesses the potential activity and could be explored further as an atherosclerosis inhibitory drug.

### Antiparasitic activity

The methanol extract from the whole plant of *T. crispa* exhibited 100% inhibition of *Plasmodium falciparum* growth after 72 h at a dose of 2.5 mg/mL (Najib Nik a Rahman et al., 1999). The chloroquine-sensitive strain of *P. falciparum w2* was also inhibited by the aqueous extract of the plant (IC_50_ 25 μg/mL). Bertani et al. (2005) studied the effect of aqueous extract on intra erythrocytic and intra hepatic cycle. In this experiment, Swiss female mice infected with *P. yoelii* 17X were used. At a dose of 110 mg/kg, the extract inhibited more than 50% of the parasite development (Bertani et al., 2005). Recently, Niljan et al. (2014) determined the antimalarial activity of the methanol extact of *T. crispa* in ICR mice infected with chloroquine-sensitive malaria parasite *P. berghei ANKA*. It was discovered that the crude extract of *T. crispa* exhibited inhibitory effect on the growth of plasmodium in a dose-dependent way. Rungruang and Boonmars (2009) investigated the *in vivo* antimalarial effect of the crude extract of *T. crispa*. The mice administrated with a daily dose of 80 mg/kg of the extract exhibited promising inhibitory activity against the parasite, *P. yoelii*. Though, only crude extracts of *T. crispa* have been evaluated for its antimalarial activity and no mechanism of action has been described, still these results are in favor of the traditional use of *T. crispa* as an antimalarial agent. The above- mentioned findings should motivate the researchers to investigate the antiplasmodial activity of pure compounds from *T. crispa* for further characterization of their antimalarial activity.

### Cytotoxic activity

The cytotoxic activities of different extracts of *T. crispa* had been studied. The cytotoxic activity of the aqueous crude extract of *T. crispa* stem was assessed against various human cancer cell lines like MCF-7, HeLa (Henrietta Lacks), Caov-3 (Homo sapiens ovary adenocarcinoma cell line), and HepG2. The cytotoxic effect exerted by the aqueous extract of *T. crispa* stem was comparable to cisplatin and tamoxifen, with IC_50_ values as follows: MCF-7 (IC_50_: 107 μg/mL), HeLa (IC_50_: 165 μg/mL), Caov-3 (IC_50_: 100 μg/mL), and HepG2 (IC_50_: 165 μg/mL; Amom et al., 2008). In another study, Froemming (2011) investigated the cytotoxic effect of the methanol extract of *T. crispa* on MDA-MB-231 (human breast adenocarcinoma cell line) and MCF-7 cancer cell lines. The methanol extract of *T. crispa* exhibited a dose-dependent cytotoxic effect on MDA-MB-231 and MCF-7 cancer cell lines with IC_50_ values of 44.8 and 33.8 μg/mL, respectively. Water, methanol and chloroform extracts of the whole plant exhibited dose-dependent antiproliferative activity against MCF-7, MDA-MB-231, HeLa, and 3T3 (swiss albino mouse embryo fibroblast) cells lines (Ibahim et al., 2011). The growth of human cancer cell lines including HL-60 (human promyelocytic leukemia cells), HepG2 and virus infected Hep3B was inhibited by the methanol extract of *T. crispa* stem. The methanol extract of *T. crispa* exerted its effect in a dose- and time-dependent manner (Sinchaikul et al., 2007).

Most of the *Tinospora* species studied have similar chemical classes of isolates or same chemical constituents but their reported pharmacological properties were different and some showed opposite responses. Amom et al. (2008) investigated the anti-proliferative activity of the aqueous crude extract of *T. crispa* and found that the extract exhibited moderate anti-proliferative activity on selected human cancer cell lines (IC_50_ MCF-7: 107 μg/ml, HeLa: 165 μg/ml, Caov-3: 100 μg/ml, and HepG2: 165 μg/ml). While Jagetia et al. (1998) evaluated the antineoplastic activity of *Tinospora cordifolia* in cultured HeLa cells and found that exposure of HeLa cells to 0, 5, 10, 25, 50, and 100 mg/mL of the extracts (methanol, aqueous, and methylene chloride) resulted in a dose-dependent but significant increase in cell killing, when compared to non-drug-treated controls. This effect of *Tinospora cordifolia* extracts was comparable or better than doxorubicin treatment. These reported studies are quite preliminary in nature and were only carried out *in vitro* using different cancer cell lines. These results are currently premature to address the antitumor potential of *T. crispa*. The active constituents and underlying mechanisms responsible for antitumor properties are still unknown and are needed to be discovered. Moreover, future studies validating therapeutic effect in *in vivo* model are required.

### Cardio-protective activity

*T. crispa* extracts and isolated active compounds showed effects on cardiovascular system both *in vitro* and *in vivo*. A study revealed that the crude alcohol extract from the stems of *T. crispa* caused an increase in blood pressure with a reduction in heart rate in anesthetized dogs (Mokkhasmit et al., 1971). A study conducted on the *n*-butanol extract of *T. crispa* revealed the presence of at least three different cardiovascular-active components which exerted their effect through β_2_-adrenergic receptors to cause a decrease in blood pressure, β_1_ and β_2_-adrenergic receptors to cause an increase in heart rate, α-adrenergic receptors to bring about an increase in blood pressure and heart rate, and a nonadrenergic and noncholinergic pathway to cause a decrease in MAP and heart rate (Praman et al., 2011, 2013). Bioassay guided fractionation of the n-butanol extract of the stems of *T. crispa* led to isolation of five active compounds namely adenosine, uridine, salsolinol, higenamine, and tyramine. The compounds exhibited effect on the mechanisms of blood pressure and heart rate in anesthetized, normal, and reserpinized rats. Salsolinol and adenosine decreased mean arterial blood pressure and heart rate, whereas uridine increased mean arterial blood pressure and decreased heart rate. Higenamine decreased mean arterial blood pressure and increased heart rate, moreover, tyramine increased mean arterial blood pressure and heart rate in normal rats. Salsolinol, tyramine, and higenamine acted via the adrenoreceptors, while uridine and adenosine acted via the purinergic adenosine A_2_ and P_2_ receptors to decrease blood pressure with a transitory decrease of heart rate followed by an increase. The crude extract of *T. crispa* along with the isolated compounds exerted a positive ionotropic effect on the rat isolated left atria stimulated with electrical field. Higenamine, salsolinol (at low concentrations) and tyramine acted through the adrenergic receptors to increase the force of the atrial contraction, however a high concentration of salsolinol acted secondarily by stimulating the release of acetylcholine. Adenosine and uridine acted through the purinergic pathways to cause negative ionotropic effects on the isolated left atria (Praman et al., 2013). The two isolated triterpenes, namely, cycloeucalenol and cycloeucalenone from the chloroform extract of the dried stems of *T. crispa*. Both of the isolated triterpenes further indicated mild cardiotonic effects, where cycloeucalenol showed slight increase in the right atrial contraction and initial reduction followed by 10% of sustained reduction on the left atria of the rat *in vitro* meanwhile cycloeucalenone, showed slight change on the right and left atrial contraction (Kongkathip et al., 2002). Imphanban et al. (2009) isolated an aporphine alkaloid, namely (−)-N-formylnornuciferine from the stems of *T. crispa*, which exhibited *in vitro* cardiotonic activity. Synthesis of the mixture, (±)-N-formylnornuciferine, by palladium-catalyzed coupling reaction, showed significant reduction in the force of contraction and the heart rate.

### Antinociceptive activity

The dried extract of the stem of *T. crispa* at a dose of 666 mL exhibited promising central analgesic activity (Almeida et al., 2001). However, the number of tested doses is not sufficient to highlight a dose-dependent effect. Owing to the lack of tested doses and a negative control, it is difficult to draw a conclusion from this study. Sulaiman et al. (2008) reported that the ethanol extract of *T. crispa* reduced acetic acid-induced writhes in mice in a dose-dependent manner. It was shown that the ethanol extract at a dose of 300 mg/kg exhibited higher analgesic response (92%) than 100 mg/kg of acetyl salicylic acid (81%). Further investigations are needed to provide an evidence for its traditional use against pain.

### Cytochromes inhibitory activities

Cytochromes P450 (CYPs) are the principal enzymes that catalyze the oxidative metabolism of drugs and other xenobiotics. Isoforms of CYP such as CYP3A4, CYP2D6, CYP2C9, and CYP2E1 have been reported to be involved in the metabolism. The inhibition of CYP results in unexpected adverse drug interactions due to changes in metabolic clearance of co-administered drug. A radiometric assay carried out by Usia et al. (2006a,b) against CYP3A4 and CYP2D6 revealed that *T. crispa* exhibited an inhibitory activity over 70% on the metabolism mediated by CYP3A4. To better understand the inhibitory mechanism, N-methyl-^14^C]erythromycin and [O-methyl- ^14^C]dextromethorphan were used as substrates in human liver microsomes and the activity of CYP was determined by measuring the production of ^14^C-formaldehyde. At a dose of 0.5 mg/mL, *T. crispa* methanol extract exhibited more than 30% increase in CYP3A4 inhibition (Subehan et al., 2006). These results suggest an inhibitory effect of *T. crispa* on CYP3A4 and CYP2D6. The effect of *T. crispa* on other isomers of CYP *viz* CYP2C9 and CYP2E1 also needs to be investigated to determine the potential drug-drug interactions. Moreover, further work is needed to purify and identify active constituents responsible for the inhibitory effect.

### Antidiabetic effect

The research undertaken by Noor and Ashcroft (1998) indicated that the orally administrated extract of *T. crispa* displayed significant antihyperglycaemic effect. The extract might comprise of compounds which initiated the insulin secretion by the modulation of β*-*cell Ca2+ concentration. Therefore, it can be additionally used as an antidiabetic agent for the treatment of type II diabetes. A potent *in vitro* insulinotropic activity in the human and rat islets and HIT-T15 (syrian hamster islet cells) B cells was observed after an oral administration of *T. crispa* extract (Noor et al., 1989). Sriyapai et al. (2009) studied the dry powder of *T. crispa* for hypoglycemic effect on patients with metabolic syndrome. Twice daily administration of 250 mg *T. crispa* dry powder significantly decreased fasting blood glucose from the baseline. Noipha and Ninla-Aesong, 2011) indicated that the extract of *T. crispa* enhanced glucose uptake by in L6 myotubes which was linked to the increased levels of GLUT1 transporter, AMPKα, and PPARγ transcript. Likewise, among twelve furanoditerpenoids isolated from the ethanol extract of *T. crispa*, borapetosides A, and borapetosides C showed hypoglycemic effect in ICR diabetic mice. These compounds reduced plasma glucose levels in normal and streptozotocin-induced type-1 diabetic mice (Lam et al., 2012). In addition bropetoside C increased glucose utilization in peripheral tissues and decreased hepatic gluconeogenesis, thus accounting for the hypoglycemic effect. Ruan et al (2012) studied the molecular mechanism of borapetoside C for hypoglycemic effects in normal and diabetic mice. The findings of the study revealed that hypoglycemic effect of borapetoside C was mediated via insulin receptor, protein kinase and glucose transporter-2 pathway. They witnessed that borapetoside C increased the glycogen level in skeletal muscle. Borapetoside C increased the expression of glucose transporter-2 as well as phosphorylation of insulin receptor and protein kinase B. Borapetoside A increased the glycogen level in skeletal muscle C2C12 and human hepatocellular carcinoma Hep3B cell lines (Ruan et al, 2013). The report suggested that borapetoside A exerted its hypoglycemic effect, primarily via augmentation of glucose utilization of skeletal muscle and liver. Borapetoside A exerted its hypoglycemic action by the stimulation of insulin receptor, protein kinase and glucose transporter-2 pathway, and the suppression of phosphoenolpyruvate carboxykinase enzymes which regulate hepatic gluconeogenesis and glucolysis (Pilkis and Granner, 1992). The borapetol B, isolated from the methanol and water extracts of *T. crispa* stem showed anti-diabetic activity in normoglycemic wistar and spontaneously type 2 diabetic Goto-Kakizaki rats. The blood glucose levels were significantly decreased by borapetol B at a dose of 10 μg/100 g in normoglycemic and in type 2 diabetic rats, while the insulin level was significantly increased. Borapetol B dose-dependently stimulated the secretion of insulin from pancreatic islets isolated from rats without damaging islet beta cells (Lokman et al., 2013). Taken together, these results support the traditional use of *T. crispa* as an antidiabetic agent. Although *T. crispa* extract and isolated pure compounds have exhibited antidiabetic activity both *in vitro* and *in vivo*, the function in humans is still unconvincing as humans were not involved in those studies. Hence, *T. crispa* is worthwhile to be considered in human diabetes treatment and, therefore, should be extensively studied.

### Clinical trials

A randomized double blind placebo controlled trial was carried out to investigate the efficacy of *T. crispa* as an additional treatment in patients with type 2 diabetes mellitus who refused insulin injection and did not respond to oral hypoglycemic drugs (Sangsuwan et al., 2004). Twenty patients were apportioned to receive *T. crispa* powder in capsule form at a dose of 1 g thrice daily for 6 months. Twenty patients received a placebo. The main results were alterations in glycosylated hemoglobin, insulin, and fasting plasma glucose levels. The baseline features of the patients in both groups were not considerably different. There were no significant alterations in glycosylated hemoglobin, insulin and fasting plasma glucose levels between the patients within the group as well as between groups. Two patients who received *T. crispa* exhibited noticeable rise of liver enzymes that reverted to normal after withdrawing *T. crispa*. Furthermore, patients in the *T. crispa* group had noteworthy weight decrease and cholesterol elevation while taking *T. crispa*. It is hence concluded that there is no proof to support the use of *T. crispa* 3 g a day for additional therapy in patients with type 2 diabetes mellitus that refused insulin injection and did not respond to oral hypoglycemic drugs. The patients receiving *T. crispa* might have a greater risk of hepatic dysfunction. Currently, only one study related to antipyretic effect of *T. crispa* could be found on http://www.clinicaltrials.gov/. In this double blind, interventional, randomized, and placebo controlled phase II trial, safety, acceptability and effectiveness of *T. crispa* extract will be determined in patients with body temperature 37.8–38.5°C. The patients will receive 500 mg of *T. crispa* extract after every 4–6 h. Besides, the efficacy and safety results of *T. crispa* extract will also be compared with those of acetaminophen. Therefore, this interesting study may lead to insightful development of knowledge regarding its clinical efficacy. Nonetheless, more operationally thorough randomized controlled trials are required.

## Toxicology

Although several studies have assessed the pharmacological properties of *T. crispa*, few data are available concerning its toxicity. Chavalittumrong et al. (1997) carried out studies to determine the acute and chronic toxicity of *T. crispa*. The acute toxicity study revealed that the ethanol extract of *T. crispa* stem did not cause any signs of toxicity or animal death at a dose of 4.0 g/kg of body weight (g/kg BW). However, the chronic toxicity test for 6 months exhibited that administration of the ethanol extract at a dose of 9.26 g/kg BW/day to rats caused hepatic and renal toxicities. Histopathological examination revealed higher frequency of bile duct proliferation and focal liver cell hyperplasia. Significant rise in alkaline phosphatase (ALP), alanine aminotransferase (ALT) creatinine levels and relative liver weights was also witnessed.

Kadir et al. (2011) reported that oral administration of the ethanol extract of *T. crispa* at doses of 100 and 200 mg/kg for 8 weeks potentiated the thioacetamide induced hepatotoxicity in rats. Moreover, they reported that the ethanol extract of *T. crispa* contained certain hepatotoxins which may be responsible for this effect (Kadir et al., 2011). A human hepatotoxicity case was reported due to chronic over use of herbal preparation of *T. crispa* stem as a prophylactic agent against malaria (Denis et al., 2007). Recently, Langrand et al. (2014) reported an incidence of toxic hepatitis linked with chronic use of high doses of *T. crispa*. They observed that a patient who received pellets of *T. crispa* had problem of dark urine and pale stools, linked with asthenia and right hypochondrial pain which lead to jaundice. The histopathological results also confirmed a toxic reaction. The herbal medicine was withdrawn on admission and the patient completely recovered without treatment, with normal liver function 2 months after the acute episode. The data reported about toxicity of *T. crispa* are very limited, so toxicological aspects of *T. crispa* need to be investigated comprehensively.

## Conclusions and future directions

Herein, we documented the existing phytochemistry, pharmacological properties, and application researches on *T. crispa*. The amount of experimental data evidenced rich nutrients and vast biological active substances in *T. crispa*. A peruse of available scientific references show that the traditional medical uses of *T. crispa* have been evaluated by modern pharmacological studies. *T. crispa* has the potential multiple pharmacological and therapeutic activities in the management of hypertension, lumbago, postpartum remedy, tuberculosis, hemorrhoids, wound healing, itching, muscle pain, etc., which can be explained by the presence of various terpenoids, alkaloids, lignans and nucleosides in the herb. The biological activities and chemical nature of the bioactive compounds must be of great attention for the researchers. Diterpenoid glycosides from *T. crispa* have shown promising antidiabetic activity. Further investigations on the terpenoids offer great potential upon which they can be predicted to be successful clinical trial candidates in antidiabetic therapy. Similarly, authentication of all the secondary metabolites should be performed carefully by advanced analytical techniques to approve the quality and conforming biological activity. Most of the mentioned pharmacological studies have provided some suggestive scientific evidence for its various traditional uses in fever, internal inflammation (Yokozawa et al., 2000, 2001; Kamarazaman et al., 2012a), pain (Almeida et al., 2001; Sulaiman et al., 2008), antibacterial (Zakaria et al., 2006; Chittur and Gunjan, 2012), malaria (Niljan et al., 2014), diabetes (Lokman et al., 2013; Ruan et al, 2013), and hypertension (Praman et al., 2011, 2013) as in Asian countries, especially in Malaysia, Indonesia, Philippines, China, Cambodia, and Bangladesh.

In a word, *T. crispa* has received much interest. However, future studies are necessary to address issues regarding composition of the extract, explicability of preclinical experiments, and lack of transformation of the preclinical results to clinical efficacy. As a result, *T. crispa* was still employed as folk prescription and the related health products are unpersuasive. Thereby, it is extremely important to conduct detailed investigations on the composition and pharmacological significance of medicinal plants and standardize the formulations based on ingredients. Further systematic studies are necessary to evaluate the efficacy using standardized extracts of *T. crispa*, and to identify the bioactive molecules responsible for the biological activities so that cost-effective, potential medicinal drug and health products can be developed at a large scale. Also, attempts should be made to conduct serious randomized human trials and determine modes or mechanisms of action, bioavailability, pharmacokinetics, and physiological pathways for specific bioactives of *T. crispa* which might be responsible behind the protective effects offered by extracts rich in flavonoids and terpenoids in many pharmacological studies. As more scientific evidences on therapeutic effects of *T. crispa* will be found, products (e.g., health care products) based on it might boom in the future.

## Author contributions

Concept, Editing, Final Approval IJ participated in the concept, editing and gave the final approval of the final version of the manuscript to be submitted for publication. WA drafted the manuscript and SB was involved in the editing process.

### Conflict of interest statement

The authors declare that the research was conducted in the absence of any commercial or financial relationships that could be construed as a potential conflict of interest.

## Abbreviations

*Tinospora crispa* (L.) Hook. f. & Thomson: *T. crispa*
LPS: lipopolysaccharide
ICAM: intracellular cell adhesion molecule
M-CSF: macrophage colony stimulating factor
VCAM: vascular cell adhesion molecule
MCP: Monocyte chemoattractant protein
AChE: acetylcholinesterase
MRSA: methicillin-resistantstaphylococcus aureus
NO: nitric oxide
DPPH: 2,2-diphenyl-1-picrylhydrazyl
FRAP: fluorescence recovery after photobleaching
BHT: butylhydroxytoluene
ICR: institute for cancer research;
IC_50_: half maximal inhibitory concentration
CYP3A4: cytochrome P450 3A4
GLUT 1: glucose transporter 1
AMPK: adenosine monophosphate-activated protein kinase
PPAR: peroxisome proliferator-activated receptor
BW: body weight
ALP: alkaline phosphatase
ALT: alanine aminotransferase.
